# First person – Emily Fackler

**DOI:** 10.1242/bio.062330

**Published:** 2025-11-17

## Abstract

First Person is a series of interviews with the first authors of a selection of papers published in Biology Open, helping researchers promote themselves alongside their papers. Emily Fackler is first author on ‘ Elucidating the genetic architecture of migratory timing in a songbird migrant, the great reed warbler, *Acrocephalus arundinaceus*’, published in BiO. Emily conducted the research described in this article while an undergraduate researcher in Dr Robert Fitak's lab at the University of Central Florida, USA. Emily is now a conservation and science technician for Disney's Animals, Science and Environment, investigating the overlap between the fields of genetics and animal behaviour.


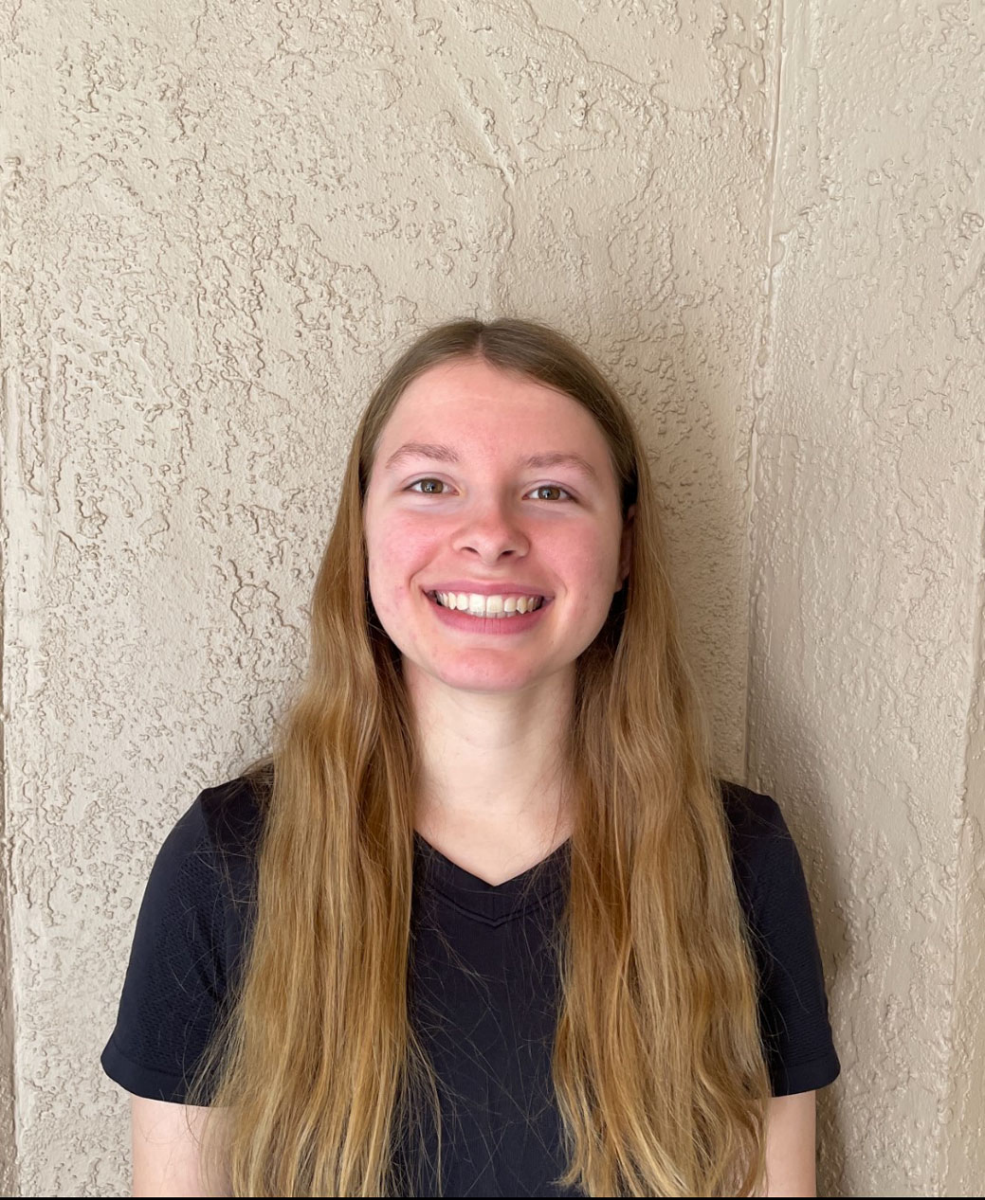


**Emily Fackler**

**Describe your scientific journey and your current research focus**

My scientific journey started by studying the behaviour of North American river otters at my local AZA (Association of Zoos and Aquariums)-accredited zoo. Ever since that study, I knew that my future was in animal behaviour. At the start of my sophomore year at the University of Central Florida, I joined the Fitak Integrative and Genomics Laboratory where I studied mating behaviour in lone star ticks (*Amblyomma americanum*), the MHC genes in green sea turtles (*Chelonia mydas*), and how genes influence migratory behaviour in great reed warblers (*Acrocephalus arundinaceus*). These research experiences allowed me to expand upon my knowledge of genomic techniques and further confirmed my passion for animal behaviour. After graduating from the University of Central Florida with my bachelor's degree in biology, I joined Disney's Animals, Science and Environment as a conservation and science technician, where I continue to study animal behaviour.

**Who or what inspired you to become a scientist?**

I've always loved science because there are so many unanswered questions. My desire to learn new things led me down the path to being a scientist and I can't imagine doing anything else.

**How would you explain the main finding of your paper?**

Each migratory season, great reed warblers show differences as to when they start their migration journey. Some migrate early, some late, and others at an intermediate time. These differences are called migratory chronotypes. Previous research has found that certain genes influence a bird's migratory chronotype; however, until now, no research has investigated whether these or similar genes influence great reed warbler migratory chronotype. After investigating 54 great reed warblers from the Czech population, we found that there are 93 genes that differ significantly between early and late chronotype individuals. These genes are related to a variety of different processes such as the breakdown of lipids and gene expression. We also found that spring migratory timing in great reed warblers has a larger genetic contribution than autumn migratory timing.

genes play a role in determining migratory timing in great reed warblers

**What are the potential implications of this finding for your field of research?**

This research provides further insight into the complex phenomenon that is migration. Our paper shows that genes play a role in determining migratory timing in great reed warblers. With habitat loss and environments changing due to climate change, it is essential that we continue to explore the genetics behind migration timing. Understanding variation in the genes involved in migration timing could offer valuable insights into how the species might adapt to changing environmental conditions in the future.

**Figure:**
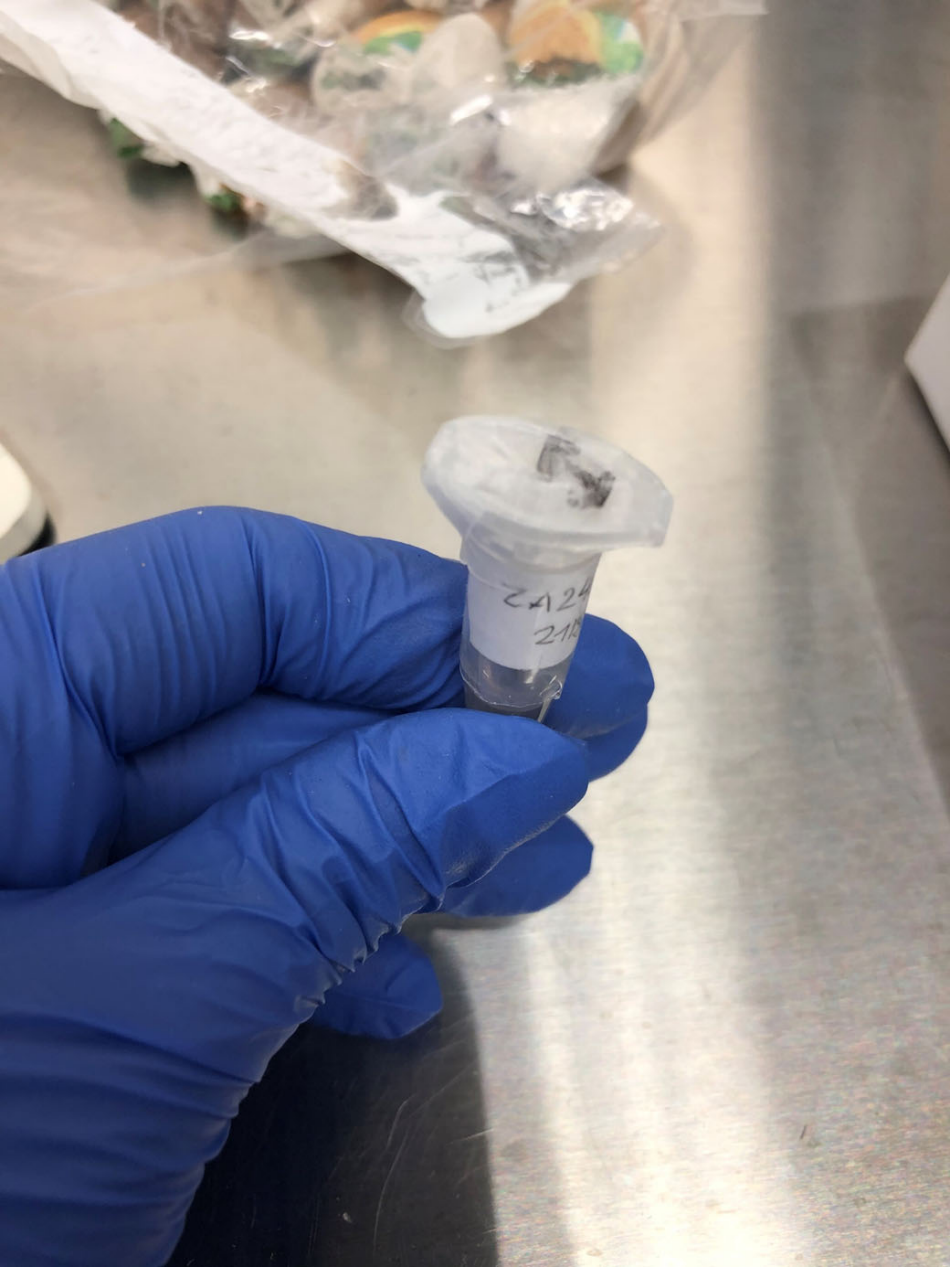
A great reed warbler blood sample prior to DNA extraction.

**Which part of this research project was the most rewarding?**

The most rewarding part of this project was being able to collaborate with some incredible scientists as well as provide new insight into an ever-growing and fascinating field of study.

**What do you enjoy most about being an early-career researcher?**

The best part about being an early-career researcher is that there is so much to learn. You are constantly asking questions, learning new things, and meeting new people.

**What piece of advice would you give to the next generation of researchers?**

A piece of advice for the next generation of researchers is to never stop asking questions. Asking questions is how you grow and learn more about the world around you.

**What's next for you?**

I have been a biologist for Disney's Animals, Science and Environment for about a year and I am extremely excited to continue to grow as a scientist as I conduct animal behaviour studies and continue learning.
